# Enhanced Antibacterial Activity of Sodium Titanate/Graphene Quantum Dot Self-Supporting Membranes via Synergistic Photocatalysis and Physical Cutting

**DOI:** 10.3390/ma18081844

**Published:** 2025-04-17

**Authors:** Shuling Shen, Ji Wang, Yaru Li, Xinjuan Liu, Zhihong Tang, Huixin Xiu, Jing Li, Guanglei Zhou

**Affiliations:** 1 School of Materials and Chemistry, University of Shanghai for Science and Technology, Shanghai 200093, China; slshen@usst.edu.cn (S.S.);; 2 Academy of Forensic Science, Shanghai 200063, China

**Keywords:** graphene quantum dots, sodium titanate, self-supporting membranes, photocatalytic antibacterial, physical cutting

## Abstract

Graphene quantum dots (GQDs) show significant promise as antibacterial agents, but their application is hindered by several limitations, including potential cytotoxicity at high concentrations, as well as concerns regarding aggregation and reusability. In this study, sodium titanate (NTO) ultralong nanotubes were utilized as both a photocatalyst and support for GQDs. The NTO/GQDs heterojunction was formed by embedding GQDs nanoplates onto the walls of NTO nanotubes. This integration significantly improved the visible light absorption and enhanced the separation and transfer of electron–hole pairs, leading to an efficient photocatalytic antibacterial process. The NTO/GQD-8 self-supporting membrane composed of these ultralong nanotubes demonstrated outstanding antibacterial efficiency (99.99%) against *E. coli* and exhibited remarkable cycling stability. Radical scavenging experiments revealed that ∙OH and e^−^ were the primary reactive species driving the photocatalytic antibacterial process. Notably, NTO and NTO/GQDs-8 exhibited distinct antibacterial outcomes. After photocatalytic treatment with NTO/GQDs-8, *E. coli* cells were completely fragmented, with no intact cell structures remaining due to the synergy effect of GQDs’ physical cutting during photocatalytic treatment.

## 1. Introduction

Photocatalytic antimicrobials have emerged as a promising alternative to traditional antibiotics and metal-based antibacterial nanomaterials due to their broad-spectrum antibacterial activity, minimal risk of antimicrobial resistance, and environmental sustainability [1,2,3]. Titanates, as derivatives of TiO_2_, demonstrate exceptional photostability, non-toxicity, and strong photocatalytic activity, making them highly suitable for photocatalytic antibacterial applications [4,5,6,7]. However, the wide band gap of titanate requires UV light for activation, and the high recombination rate of electron–hole pairs results in low photocatalytic efficiency. To address these limitations, researchers have employed various techniques, such as incorporating Ag and Au nanoparticles as co-catalysts. By combining the SPR effect, carrier separation ability, and inherent antibacterial properties of Ag, sodium titanate (NTO) have exhibited remarkable bactericidal activity under both dark and light conditions [8]. However, the use of Ag and Au is limited by their status as precious metals, making them costly, and their potential toxicity to human cells, animals, and ecosystems. These issues highlight the need for alternative materials. Recent advances in graphene-based photocatalysts have achieved notable progress [9,10,11]. However, these systems still suffer from limitations: the strong π–π interactions between graphene layers reduce accessible surface area and poor interfacial charge transfer due to weak van der Waals interaction.

Graphene quantum dots (GQDs) are small fragments of graphene, typically less than 20 nm in size, which impart unique optical electronic properties and biocompatibility [12,13,14,15]. With the advantage of their small size, high surface area, and low toxicity, GQDs, as graphene derivatives, also exhibit antimicrobial activities by interacting with bacterial cell membranes, leading to structural damage and cell leakage, which results in bacterial death [16,17,18]. Numerous studies have established this physical cutting phenomenon as a predominant mode of antimicrobial activity characteristic of graphene family materials [17,19,20]. Extensive research has demonstrated that the “nanoknife” effect depends critically on size, edge sharpness, and surface properties. Molecular dynamics simulations have shown that smaller graphene sheets (<5 nm) exhibit enhanced membrane penetration capability [21], while recent studies emphasize that antibacterial efficacy strongly correlates with edge density and defect sites [22]. Additionally, the strong absorption across the range of the UV-visible spectrum and high electrical conductivity make GQDs an ideal co-catalyst for enhancing the photocatalytic antimicrobial activity of photocatalysts [23,24]. Lei and coworkers revealed that GQDs not only extended the light absorption range to longer wavelengths, but also acted as an electron acceptor instead of a photosensitizer to enhance the photocatalytic performance [25]. However, the practical applications of GQDs powders in antibacterial applications faces challenges, including difficulties in recovery and reusability, potential environmental contamination, and aggregation issues.

In this study, GQDs were employed as co-catalysts to replace precious metal nanoparticles, and ultralong NTO nanotubes/GQDs were synthesized by the stirring hydrothermal method. The 0D GQDs were uniformly anchored onto the walls of 1D NTO nanotubes. This configuration not only substantially enhanced visible light absorption and facilitated the separation of photogenerated charge carriers, but also enabled the GQDs to function as “nanoknives”. The ultralong 1D nanotubular architecture of NTO serves three critical functions: (1) providing pathways for electron transport, (2) offering an ideal support for dense GQDs anchoring, while effectively preventing their aggregation, and (3) enabling direct fabrication of self-supporting photocatalytic membranes that improving catalyst recovery and reuse. The NTO/GQD self-supporting membranes achieved an antimicrobial rate of 99.99% against *Escherichia coli* (*E. coli*) under visible light. This study highlights the dual role of GQDs in enhancing the photocatalytic activity of NTO and physical cutting of *E. coli* cells to achieve highly efficient antibacterial performance.

## 2. Materials and Methods

### 2.1. Materials

TiO_2_ powder was purchased from Deguassa Co. Ltd., Frankfurt, Germany. Aphanitic graphite was purchased from Chenzhou Botai Graphite Co., Ltd., Chenzhou, China. Sodium nitrate (NaNO_3_), concentrated sulfuric acid (H_2_SO_4_), potassium permanganate (KMnO_4_), hydrogen peroxide (H_2_O_2_), hydrochloric acid (HCl), sodium hydroxide (NaOH), triethanolamine (TEOA), and isopropyl alcohol (IPA) were purchased from Sinopsin Chemical Reagent Co., Ltd., Shanghai, China. P-benzoquinone (BQ) was purchased from Shanghai Titan Technology Co., Ltd., Shanghai, China. Sodium chloride (NaCl) and potassium bromate (KBrO_3_) were sourced from Aladdin Reagents Ltd., Shanghai, China. The *E. coli* strains utilized in the experiment were obtained from Shanghai Luwei Technology Co., Ltd., Shanghai, China.

### 2.2. Preparation of GQDs

GQDs were prepared using a method previously developed by our team [12]. Typically, 5 g of aphanitic graphite and 2.5 g of NaNO_3_ were mixed with 115 mL of H_2_SO_4_, and the mixture was sonicated for 25 min at a low temperature (below 10 °C). Slowly, 15 g of KMnO4 was added to the mixture, during which the solution gradually turned dark green. The reaction solution was then heated to 35 °C and maintained at this temperature for 45 min, resulting a color change from dark green to earthy yellow. After cooling to room temperature, H_2_O_2_ was added to eliminate excess KMnO_4_, followed by the addition of HCl to remove the residual H_2_O_2_. The solution was subsequently washed with deionized water until a neutral pH of 7 was achieved. After 2 h of ultrasonication, the GQD solution was obtained through centrifugation.

### 2.3. Preparation of NTO/GQDs

NTO/GQDs were prepared by a stirring hydrothermal method [26,27,28]. Taking the sample with 8 wt% of GQDs in NTO/GQDs as an example, the following procedure was performed: 12 g of NaOH was weighed and slowly mixed with 20 mL of deionized water. The mixture was stirred until it was completely dissolved. Subsequently, 0.2 g of TiO_2_ powder was added to the NaOH solution and stirred for 5 min. The pre-prepared GQDs solution was added at a ratio of 8 wt%, followed by the addition of deionized water to bring the total solution volume to 30 mL. The resulting mixture was transferred to a 50 mL autoclave. The hydrothermal reaction was conducted at 130 °C for 24 h with a stirring speed of 300 rpm. After the reaction, the reactor was naturally cooled to room temperature. Finally, the product was washed with deionized water until a neutral pH of 7 was achieved. The product was transferred to a sealed brown sample bottle, and defined as NTO/GQDs-8. Similarly, samples containing 4 wt% and 6 wt% of GQDs were named as NTO/GQDs-4 and NTO/GQDs-6, respectively.

Pure NTO ultralong nanotubes, used as the control sample, were synthesized following a similar procedure, except that GQDs were not added during the preparation process.

### 2.4. Preparation of NTO/GQD Self-Supporting Membranes

NTO/GQD ultralong nanotubes and pluronic F-127 (0.1 wt%) were mixed dispersed in 40 mL of deionized water using ultrasonication for 10 min. The resulting uniform dispersion was filtered through a cellulose acetate membrane. The wet membrane was then freeze-dried, resulting in the final NTO/GQD self-supporting membrane.

### 2.5. Characterization

The morphology of the samples was characterized using a field emission environmental scanning electron microscope (SEM, Hitachi S4800, Tokyo, Japan) with an accelerating voltage of 30 kV. The morphology, lattice fringes, and elemental mapping of specific regions of the samples were analyzed using a FEI Tecnai G2 F30 transmission electron microscope (TEM, FEI, Hillsboro, OR, USA). The crystal phases of the samples were identified with a Bruker D8 Advanced powder X-ray diffractometer (XRD), employing a Cu-Kα X-ray source (λ = 0.15418 nm). The scanning range was set to 5–80°, with a scanning speed of 2°·min^−1^. Functional groups present in the samples were determined using a Perkin-Elmer Spectrum 100 Fourier transform infrared (FTIR) spectrometer (Waltham, MA, USA). The scanning range was 450–4000 cm^−1^ at a resolution of 1 cm^−1^. The elemental composition, chemical valence states, and valence band positions of the samples were determined using a Thermo Fisher Scientific EscaLab 250Xi X-ray photoelectron spectrometer (XPS) (Waltham, MA, USA). The UV-vis absorption spectra of the samples were measured in the range of 250–800 nm using a Lambda 750 UV/Vis/NIR spectrophotometer (Perkin-Elmer, Waltham, MA, USA). The photoluminescence (PL) spectra were examined using an F-7000 fluorescence spectrometer (Hitachi, Tokyo, Japan), with an excitation wavelength of 325 nm and a scanning range of 350–700 nm.

All electrochemical tests for the samples were conducted using a Gamry three-electrode electrochemical workstation, where Pt served as the counter electrode, an Ag/AgCl electrode was used as the reference electrode, and a 0.5 M Na_2_SO_4_ solution was employed as the electrolyte. The preparation method for the working electrode was as follows: 2 mg of the sample was placed into a 1.5 mL centrifuge tube, to which 20 µL of Nafion solution and 180 µL of anhydrous ethanol were added. The mixture was sonicated for 30 min at room temperature to form a uniform slurry. Then, 100 µL of the slurry was drop-cast onto FTO conductive glass with an effective area of 1 × 1 cm^2^. The electrode was allowed to dry naturally for 12 h. The electrochemical impedance spectroscopy (EIS) of the sample was measured in the frequency range of 10 MHz to 10 mHz.

### 2.6. Photocatalytic Antibacterial Test of NTO/GQD Self-Supporting Membranes

Gram-negative *E. coli* was chosen as the test organism. The bacteria were cultured in 10 mL of Luria–Bertani (LB) broth at 37 °C for 6 h. To prepare the inoculum, the bacterial suspension was serially diluted using 0.9% NaCl saline solution. Following the protocol specified in GB/T 31402-2023 [29], the outer surface of the membrane sample was used as the test area. A 200 µL aliquot of the bacterial suspension was evenly spread over the surface of the sample and covered with a polyethylene (PE) film, ensuring full contact between the bacterial solution and the membrane without any leakage.

The sample was placed under an LED lamp (Beijing Perfectlight (Beijing, China): PLS-LED 100C, λ = 420 nm, 110 W); the distance between the light source and sample was fixed at 15 cm (39 mW/cm^2^). After irradiation, the samples were thoroughly rinsed with 0.9 wt% saline solution to collect the bacterial suspension. Subsequently, 300 µL of the collected bacterial suspension was evenly spread onto a culture medium and incubated at 37 °C for 24 h. The bacterial colonies formed on the petri dish were then counted, and the bactericidal rate was calculated. Meanwhile, the control group was kept in a constant temperature incubator at 37 °C. The antibacterial rate for each sample was calculated according to Equation (1):

(1) $$R=\frac{N_{0}-\;N_{x}}{N_{0}}\times 100\%$$

where *R* represents antibacterial rate, *N*_0_ refers to the number of colonies growing on the medium corresponding to the blank control, and *N_x_* refers to the number of colonies growing on the medium corresponding to the samples.

## 3. Results and Discussion

**Figure 1:**
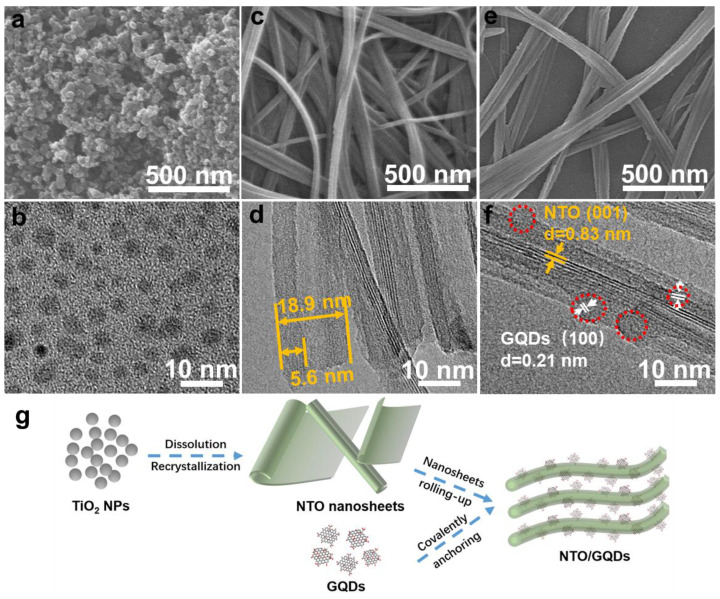
(**a**) SEM image of TiO_2_ and (**b**) TEM image of GQDs. (**c**) SEM image and (**d**) HRTEM image of NTO. (**e**) SEM image and (**f**) HRTEM image of NTO/GQDs-8. The red dashed circles highlight the locations of GQDs. (**g**) Schematic illustration for the synthetic process of NTO/GQDs.

Figure 1a presents the SEM image of TiO_2_, which reveals uniformly sized nanoparticles with an average diameter of ~25 nm. The particle size distribution of GQDs (Figure 1b) shows that the average diameter of GQDs is about 4.5 nm. By using these TiO_2_ and GQDs as precursors, NTO/GQDs were synthesized by the stirring hydrothermal method under a high concentration of NaOH. The SEM image of NTO in Figure 1c displays an ultralong one-dimensional structure extending to several microns in length. HRTEM image confirm that these structures are ultralong nanotubes with an approximate diameter of 18.9 nm and a wall thickness of around 5.6 nm (Figure 1d). These results indicate that the ultralong nanotubes are formed following the alkali heat treatment of TiO_2_ [26,27,28]. The SEM image (Figure 1e) and HRTEM image (Figure 1f) of NTO/GQDs show that the ultralong nanotube structure remains intact. Additionally, GQDs are uniformly distributed on the wall of NTO nanotubes. The lattice spacing of 0.83 nm corresponds to the NTO (001) crystal plane, while the spacing of 0.21 nm and 0.39 nm corresponds to the (100) and (002) crystal planes of the graphene structure, respectively [30,31]. The formation mechanism of the NTO/GQD composite, as illustrated in Figure 1g, involves multiple steps under hydrothermal conditions (10 M NaOH, 130 °C): TiO_2_ NPs completely dissolve in an alkaline solution, which subsequently recrystallized into lamellar NTO nanosheets. The surface energy drives the rolling-up of NTO nanosheets into nanotubes [26]. Simultaneously, GQDs covalently anchor onto the NTO surface.

**Figure 2:**
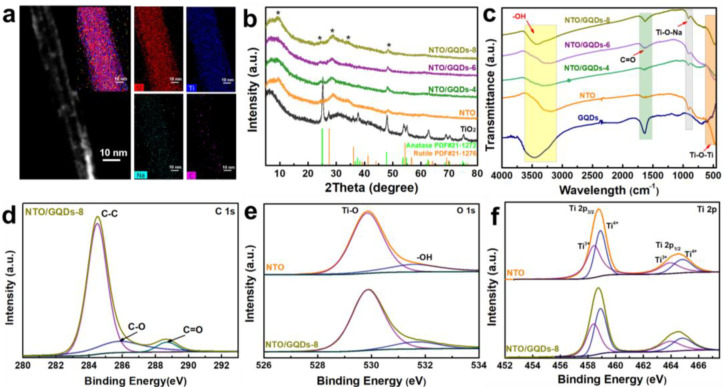
(**a**) STEM images of NTO/GQDs-8 with corresponding EDS element mapping. (**b**) XRD patterns (The asterisks (*) denote characteristic diffraction peaks of NTO), (**c**) FTIR spectra of TiO_2_, GQDs, NTO, and NTO/GQDs with varying GQD loadings. (**d**–**f**) XPS spectra of NTO and NTO/GQDs-8.

Figure 2a shows the STEM images of NTO/GQDs, along with the corresponding EDS element mapping, which confirms the presence of O, Ti, Na, and C elements in the sample. The C element was observed to be both aggregated and dispersed alongside O, Ti, and Na, further supporting the incorporation of GQDs onto the NTO nanotubes. The GQDs were uniformly distributed along the extension direction of the nanotubes. These findings confirm the successful loading of GQDs onto NTO ultralong nanotubes.

Figure 2b shows the XRD patterns of TiO_2_, NTO, and NTO/GQDs. The results indicate that the TiO_2_ powder primarily consists of the anatase phase of TiO_2_ (PDF#21-1272), with a minor presence of the rutile phase of TiO_2_ (PDF#21-1276). After hydrothermal treatment with NaOH, the characteristic diffraction peaks of TiO_2_ disappear, and new diffraction peaks at 9.56°, 25.27°, 28.97°, 34.2°, and 48.39° emerge. These peaks can be indexed to the (001), (011), (300), (−203), and (020) crystal planes of Na_2_Ti_3_O_7_ (PDF#31-1329) [30,32,33]. To investigate the effect of GQDs on the structure of NTO nanotubes, XRD analysis was also performed for NTO/GQDs-4, NTO/GQDs-6, and NTO/GQDs-8. The characteristic peaks of GQDs were not detected in the composites. The absence of detectable GQDs peaks in the composite XRD pattern can be attributed to three key factors: the ultra-small size of GQDs (4.5 nm) results in significant peak broadening due to the Scherrer effect, making their diffraction features inherently weak and difficult to distinguish (Figure S1); the relatively low content of GQDs in the composite further reduces their diffraction intensity below the detection limit of conventional XRD; and the homogeneous distribution of GQDs on the NTO nanotube surfaces prevents any localized accumulation that might otherwise enhance diffraction signals.

The surface functional groups of NTO and GQDs were analyzed using FT-IR. As shown in Figure 2c, the characteristic peak at 909 cm^−1^ for pure NTO corresponds to the stretching vibration of the Ti-O bond in the Ti-O-Na group [33,34]. Upon incorporating GQDs, the stretching vibration of Ti-O-Ti at 471 cm^−1^ shifts to higher wavenumbers, which can be attributed to the coupling between the stretching vibration peaks of Ti-O-Ti and Ti-O-C bonds. This overlap occurs because the broad Ti-O-Ti stretching region (400–800 cm^−1^) encompasses the characteristic Ti-O-C vibration (~798 cm^−1^), making their individual contributions challenging to resolve [35]. In the NTO/GQDs series samples, the stretching vibration of C=O at 1639 cm^−1^, corresponding to -COOH-rich GQDs, becomes more pronounced with an increasing GQD content. Furthermore, the incorporation of GQDs causes the stretching vibration peak of -OH at 3471 cm^−1^ to shift to higher wavenumbers. This shift is attributed to the combined contributions of -OH groups from both adsorbed water molecules (H_2_O) and the surface -OH groups of GQDs.

The composition of the samples and the chemical state of the elements were further analyzed using XPS. The C 1s XPS spectrum of NTO/GQDs-8 in Figure 2d was deconvoluted into three peaks at 284.50 eV, 286.01 eV, and 288.70 eV, corresponding to C-C, C-O, and C=O bonds, respectively, all originating from the GQDs. The O 1s XPS spectrum of NTO shows two characteristic peaks: 529.86 eV (lattice oxygen), and 531.70 eV (O-H in adsorbed H_2_O or GQDs) [36,37]. Moreover, the increased intensity of the O-H peak in NTO/GQDs-8 suggests an interaction between NTO and the hydroxyl groups on the surface of GQDs [38]. The Ti 2p XPS spectra of both NTO and NTO/GQDs-8 exhibit four peaks. The peaks at 458.47 eV and 463.93 eV are assigned to Ti^3+^, while those at 458.90 eV and 464.84 eV correspond to Ti^4+^. The presence of Ti^3+^ species in both NTO/GQDs and pure NTO confirms their intrinsic origin, independent of GQD-mediated reduction. Hydrothermal conditions spontaneously induce Ti^4+^ reduction through synergistic thermal and pressure [39]. These results indicate that the integration of GQDs not only preserves the structural integrity of NTO, but also enhances surface interactions.

**Figure 3:**
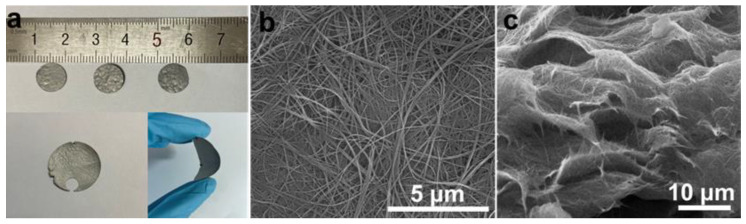
(**a**) Digital images and SEM images of (**b**) the surface and (**c**) the longitudinal cross-section of the NTO/GQD-8 self-supporting membrane.

Porous self-supporting membranes were prepared through the vacuum filtration of ultralong NTO nanotubes/GQDs. These membranes exhibit excellent toughness, allowing for flexibility in bending and customization into various shapes and sizes, as shown in Figure 3a. This adaptability makes them highly suitable for diverse photocatalytic antibacterial applications. To examine the morphology and formation process of NTO/GQD self-supporting membranes, SEM analysis was performed on both the surface and longitudinal cross-section of the membranes. During the vacuum filtration process, the ultralong NTO nanotubes/GQDs are arranged in a staggered pattern along the horizontal direction (Figure 3b). The horizontally interwoven layers stack under the combined effects of gravity and vacuum filtration, resulting in the formation of porous self-supporting membranes (Figure 3c). The excellent toughness of the self-supporting membrane is primarily derived from the three-dimensional network formed by NTO/GQD ultralong nanotubes and the mechanical advantages of the materials themselves.

**Figure 4:**
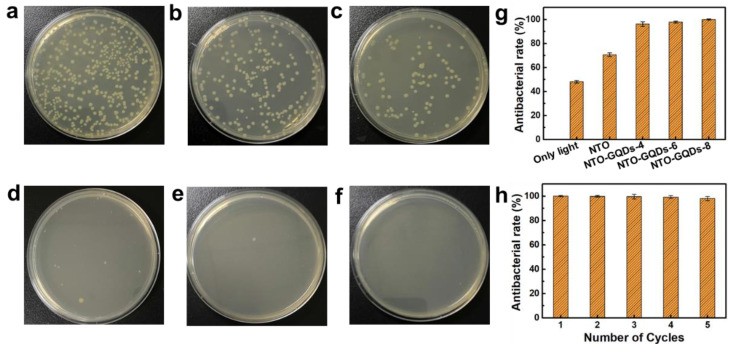
Digital images of *E. coli* after the treatment: (**a**) without catalysts and light irradiation, (**b**) without catalysts but with light irradiation, and (**c**–**f**) with NTO, NTO/GQDs-4, NTO/GQDs-6, and NTO/GQDs-8 as catalysts and light irradiation, respectively. (**g**) Antibacterial rates of different samples. (**h**) Antibacterial performance of NTO/GQDs-8 over five cycles.

The photocatalytic antibacterial activity of NTO/GQD self-supporting membranes under 420 nm LED light was evaluated using the dilution-coated plate method, with *E. coli* serving as the model bacterium. A control experiment was conducted without light irradiation or catalysts. To investigate the effect of light, *E. coli* was also treated under light irradiation without catalysts. Figure 4 shows the coated plates used in the antibacterial experiments for the NTO series self-supporting membrane. The antibacterial rates, calculated using Equation (1), are presented in Figure 4g, where *N*_0_ represents the number of bacterial colonies in the control experiment. Figure 4b demonstrates that light irradiation alone can inactivate a portion of the bacteria (48.1%). When the NTO membrane is employed as an antibacterial agent, the antibacterial rate increases to 70.6%. Although this represents a notable improvement, it is still insufficient to achieve complete antibacterial efficacy. In contrast, all NTO/GQD samples exhibit outstanding antibacterial activity, with NTO/GQDs-8 achieving an antibacterial rate of 99.99%, eliminating nearly all *E. coli*. Due to the self-supporting membrane structure of NTO/GQDs-8, it can be easily recovered and reused. After five cycles of use, the antibacterial rate of the NTO/GQD-8 self-supporting membrane remains above 98%, demonstrating excellent cyclic stability (Figure 4h).

**Figure 5:**
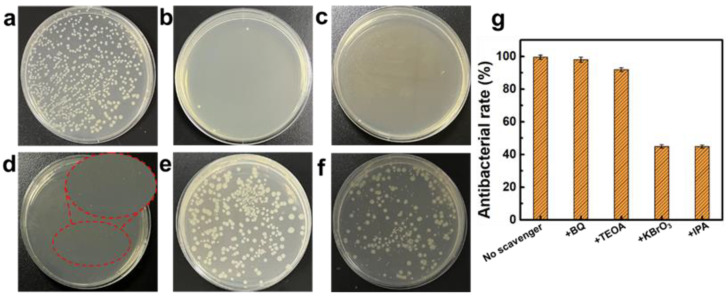
Digital images of *E. coli* treated with NTO/GQDs-8 and different trapping agents: (**a**) control, (**b**) no trapping agents, (**c**) BQ, (**d**) TEOA, (**e**) KBrO_3_, (**f**) IPA. (**g**) Antibacterial rates of NTO/GQDs-8 with different trapping agents.

To investigate the active species involved and the underlying photocatalytic antibacterial mechanism, radical scavenging experiments were firstly conducted for NTO/GQDs-8. BQ, TEOA, KBrO_3_, and IPA were employed as scavengers for ∙O^2−^, h^+^, e^−^, and ∙OH, respectively. The trapping agents were used at a concentration of 5 mM, which, as demonstrated in previous studies [40,41], does not affect the growth of *E. coli*. Figure 5 presents the coated plates and antibacterial rates of NTO/GQDs-8 in the presence of different trapping agents. The results indicate that the photocatalytic antibacterial rates are not significantly affected when BQ and TEOA are added to the reaction system, suggesting that O^2−^ and h^+^ are not the main active species. In contrast, the addition of KBrO_3_ and IPA led to a notable decrease in the photocatalytic antibacterial rate, indicating that ∙OH and e^−^ are the primary reactive species involved in the photocatalytic antibacterial process of NTO/GQDs-8.

**Figure 6:**
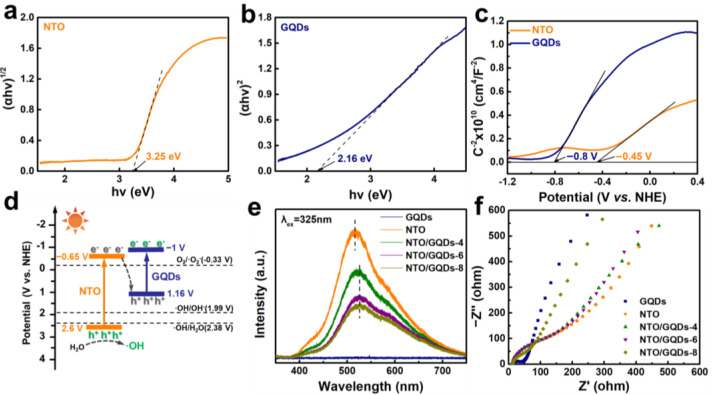
Tauc plots of (**a**) NTO and (**b**) GQDs. (**c**) Mott–Schottky plots and (**d**) schematic illustration of the S-scheme charges transfer mechanism in the NTO/GQDs heterojunction system. (**e**) PL spectra and (**f**) EIS plots of NTO, GQD, and NTO/GQD samples.

To gain a deeper insight into the enhanced photocatalytic antibacterial mechanism of NTO/GQDs under visible light, UV-Vis absorption spectra and Mott–Schottky (MS) curves were measured to construct an energy band model for NTO/GQDs. Figure 6a and Figure 6b present the Tauc plots of NTO and GQDs, respectively, which were calculated using the Kubelka–Munk formula according to UV-Vis absorption spectra (Figure S1). The results show that the band gaps (*E_g_*) of NTO and GQDs are 3.25 eV and 2.16 eV, respectively. Clearly, pure NTO cannot response for visible light. But after the decoration of GQDs, all the NTO/GQD samples exhibit enhanced visible light absorption capacity (Figure S1). The MS curves for both NTO and GQDs display a positive slope (Figure 6c), indicating their typical n-type semiconductor behavior [42,43,44]. The intersection of the tangent line with the *x*-axis occurs at −0.8 V and −0.45 V (vs. NHE), respectively. For n-type semiconductors, the flat band potential is approximately 0.2 V more negative than the conduction band potential (*E_CB_*). Thus, the *E*_CB_ positions of GQDs and NTO are estimated to be −1.0 V and −0.65 V, respectively. Using Equation (2), the valence band positions (*E_VB_*) of GQDs and NTO are estimated to be 1.16 V and 2.6 V, respectively.

(2) $$E_{VB}=E_{g}+E_{CB}$$

Based on these values, the band structures of NTO and GQDs are illustrated in Figure 6d. Combined with the results of scavenging experiments (Figure 5) that ∙OH and e^−^ are the primary reactive species involved in the photocatalytic antibacterial process of NTO/GQDs-8, it can be concluded that NTO/GQDs form an S-scheme heterojunction, where the electrons with stronger reducing ability in the CB of GQDs and the holes with stronger oxidizing ability in the VB of NTO are effectively preserved.

Once GQDs and NTO form an S-scheme heterojunction, it facilitates the transfer of photo-generated charge carriers and suppress the recombination of electrons and holes, thereby enhancing the catalytic activity. To verify this, the photoluminescence (PL) spectra PL and EIS tests were conducted. The excitation wavelength was 325 nm. The samples were deposited onto a glass slide and then allowed to dry, forming a solid membrane. In the PL spectrum of GQDs (Figure 6e), no distinct emission peaks were observed, which may be attributed to fluorescence quenching caused by the aggregation of GQDs [45]. The PL spectra of NTO/GQDs-8 show significant fluorescence quenching (~59% lower PL intensity than pristine NTO) and peak red-shift from 515 nm to 525 nm, which collectively demonstrate the effective charge separation and formation of interfacial states that mediate electron transfer from NTO to GQDs [46,47,48,49]. The EIS Nyquist plot of GQDs displays the smallest Nyquist radius, indicating fast electron movement in GQDs due their nature of graphene (Figure 6f). Similarly, NTO/GQDs-8 exhibits the smallest Nyquist radius among all NTO/GQD samples, suggesting the lowest interfacial transfer resistance between NTO and GQDs in NTO/GQDs-8. The collective experimental evidence—including the distinct PL quenching, characteristic red shift, and reduced charge transfer resistance—conclusively demonstrates the successful construction of an S-scheme heterojunction between NTO and GQDs [50,51,52].

These findings suggest that GQDs play multiple roles in enhancing photocatalytic capability. (1) Enhancing visible-light absorption: GQDs exhibit strong visible-light absorption (Figure S2). When GQDs are integrated with NTO, they significantly expand the absorption range and intensity of the composite, enabling efficient antibacterial activity even under 420 nm LED irradiation. (2) Facilitating charge separation and transfer: GQDs and NTO form an S-scheme heterojunction, which suppresses the recombination of electron–hole pairs (Figure 6e). Due to the excellent conductivity, GQDs can rapidly transfer the separated electrons to the catalyst surface for subsequent redox reactions (Figure 6f). It is worth noting that the oxidation degree of graphene-based materials is indeed a critical factor affecting nanocomposite structure and photocatalytic efficiency [53,54]. For example, oxygen-containing functional groups (e.g., C=O, C-OH) on GQDs can modulate electron transfer kinetics at the NTO/GQDs interface, where an optimal oxidation level balances efficient charge separation against recombination losses. While the degree of π-conjugation restoration in GQDs governs their light absorption characteristics, potentially extending visible-light response up to 600 nm depending on reduction conditions. Furthermore, the density of carboxyl groups significantly affects interfacial bonding with NTO surface Ti-OH groups, with moderate oxidation maximizing both heterojunction stability and preservation of active sites. Although our current hydrothermal synthesis produces GQDs with controlled reduction degrees, deliberate oxidation-state engineering through chemical treatments (e.g., HNO_3_/H_2_O_2_ oxidation or NaBH_4_ reduction) could further optimize the charge transport properties, representing a promising avenue for future performance enhancement.

**Figure 7:**
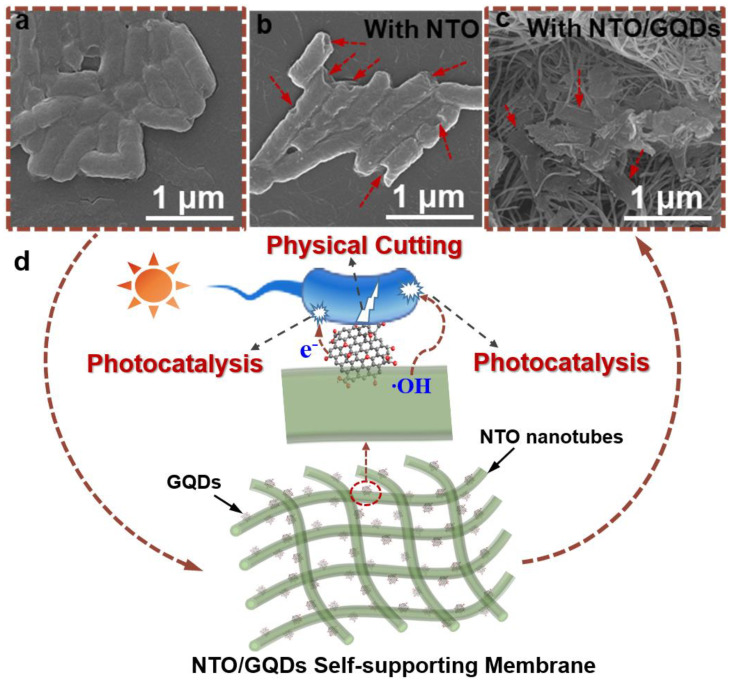
SEM images of *E. coli* cells untreated (**a**), treated with NTO (**b**), and NTO/GQDs-8 (**c**) under visible light irradiation. (**d**) Schematic illustration of the enhanced antibacterial activity of NTO/GQD self-supporting membranes through synergistic photocatalysis and physical cutting.

To further investigate the photocatalytic antibacterial mechanism of NTO/GQDs, the morphology and structure of *E. coli* cells after the treatment with NTO and NTO/GQDs-8 was observed using SEM. The morphology of untreated *E. coli* cells is smooth and plump, with intact cell walls and membranes, indicating that the bacteria are in a healthy state (Figure 7a). After the photocatalytic reaction with NTO, the morphology of *E. coli* cells underwent significant changes, as shown in Figure 7b. Almost every cell exhibits damage to its cell wall; however, these damages are localized, and the overall structure of the bacterial cells is still largely preserved. When NTO/GQDs-8 is used as the catalyst, the *E. coli* cells after photocatalytic treatment are completely fragmented, with no intact cell structures remaining. This indicates that NTO/GQDs-8 exhibits a more thorough antibacterial effect as a catalyst. Generally, at a short time of photocatalytic treatment, *E. coli* cells only deform, characterized by surface protrusions and pits (Figure 7b). With extended treatment, the cells become severely distorted and eventually fractured [55]. Therefore, the bacterial morphology in Figure 7c is not solely the result of NTO/GQDs-8 photocatalytic antibacterial action. GQDs not only enhance the photocatalytic activity of NTO, but the GQD nanoplates embedded in the NTO matrix also function as “nanoknives” that cut bacterial cells [19,20,21,22,56]. In synergy with photogenerated active oxygen species, they accelerate the destruction of bacterial cells and more effectively degrade the cell remnants, thereby reducing the toxicity of the bacterial debris (Figure 7d). As shown in Table S1, an NTO/GQD membrane displays superior antibacterial kinetics which under comparable light intensity (~40 mW/cm^2^) achieves 99.99% *E. coli* inactivation within 60 min, outperforming TiO_2_ nanotubes (>99% in 60 min) and Ag/TiO_2_ coatings (>98% in 30 min). Despite lower power density (39 vs. 100 mW/cm^2^), NTO/GQDs match the performance of Ag/TiO_2_ nanofiber membranes (99.9% in 30 min). GQDs provide dual functionality, including physical disruption via GQDs, and photogenerated ·OH and e^−^. The NTO/GQD membrane shows promise for water disinfection, air purification, protective textiles and self-sterilizing medical masks.

## 4. Conclusions

In summary, through a facile stirring-assisted hydrothermal approach, graphene quantum dots (GQDs) averaging 4.5 nm in diameter were uniformly anchored onto the surface of NTO ultralong nanotubes. The resulting NTO/GQD maintained its one-dimensional nanotubular morphology and was subsequently processed into self-supporting membrane structures. The NTO/GQD-8 self-supporting membranes demonstrated remarkable photocatalytic antibacterial performance, achieving a visible light-induced antibacterial rate of 99.99%, which is 1.42 times higher than that of pure NTO membranes. This enhancement can be attributed to the synergistic effects of photogenerated reactive oxygen species and the physical cutting action of GQDs. Additionally, the membranes exhibited excellent stability, retaining an antibacterial rate of over 98% after five cycles. This study highlights the potential of NTO/GQD self-supporting membranes as efficient, and reusable photocatalytic antibacterial agents. Furthermore, it provides valuable insights for advancing the application of photocatalytic antibacterial technologies in coatings, air purification, and water treatment.

## Data Availability

The original contributions presented in this study are included in the article/Supplementary Material. Further inquiries can be directed to the corresponding authors.

## Supplementary Materials

The following supporting information can be downloaded at: https://www.mdpi.com/article/10.3390/ma18081844/s1, Figure S1: XRD pattern of GQDs. Figure S2: UV-Vis DRS spectra of NTO, GQDs, NTO/GQDs-4, NTO/GQDs-6, and NTO/GQDs-8. Table S1: The antibacterial properties of Ti-based membrane and coating. References [57,58,59,60,61] are cited in the Supplementary Materials.
