# Supplementary material for: Enhanced Antibacterial Activity of Sodium Titanate/Graphene Quantum Dot Self-Supporting Membranes via Synergistic Photocatalysis and Physical Cutting
Source: Materials (Basel). 2025 Apr 17;18(8):1844. doi: 10.3390/ma18081844 (PMC12028856; doi:10.3390/ma18081844)
Supplement: Supplementary file 1 [file materials-18-01844-s001.zip › materials-3552778-supplementary.pdf]

Enhanced Antibacterial Activity of Sodium Titanate/Graphene Quantum Dots Self-Supporting Membranes via Synergistic Photocatalysis and Physical Cutting

Shuling Shen,[a] Ji Wang,[a] Yaru Li,[a] Xinjuan Liu,[a] Zhihong Tang,[a] Huixin Xiu,[a] Jing Li,[a]* Guanglei Zhou[b]*

[a] School of Materials and Chemistry, University of Shanghai for Science and Technology, Shanghai, 200093, China

[b] Academy of Forensic Science, Shanghai, 200063, China

*Corresponding authors: lijing6080@usst.edu.cn; zhouzhou9920736@163.com

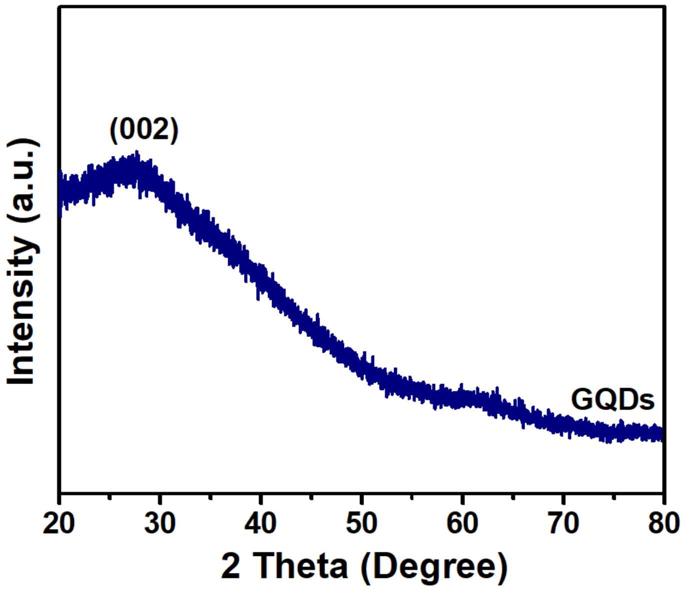

Figure S1 XRD pattern of GQDs.

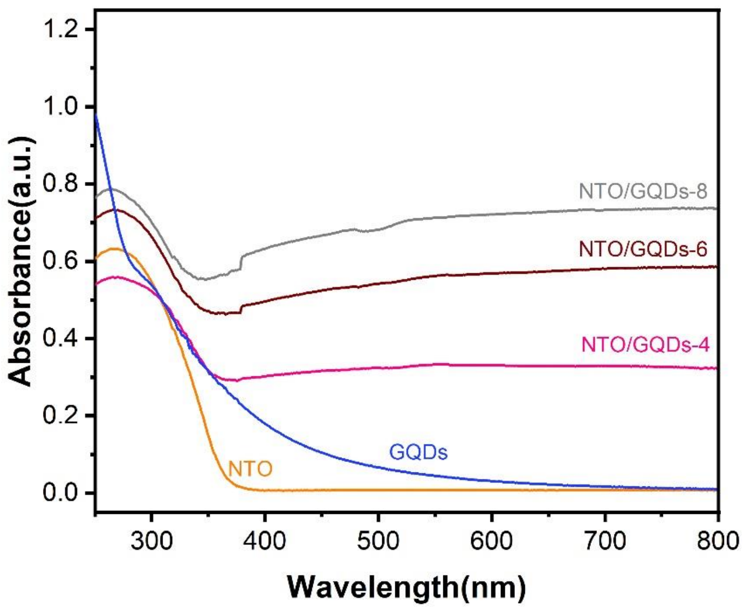

Figure S2 UV-Vis DRS spectra of NTO, GQDs, NTO/ GQDs-4, NTO/ GQDs-6, and NTO/ GQDs-8.

Table S1 The antibacterial properties of Ti-based membrane and coating.

Antibacterial agents	Light source	Antibacterial performance	Ref.
GQDs/NTO nanotube membrane	110 W LED (λ=420 nm, 39 mW/cm^2) 60 min	*E. coli,* 99.99 %	This work
TiO_2 nanotube thin films	10 W black light fluorescent lamp (λ=365 nm, 40 W/cm^2), 60 min	*E. coli*, >99%	[1]
Ag/TiO_2 coating	350 W Xe arc lamp (λ>420 nm), 30 min	*E. coli*, >98%	[2]
Al-O/Ti-O thin films	400 W mercury lamp with middle pressure (400 nm>λ>200nm), 60 min	*E. coli*, 95%	[3]
Ag/AgBr/TiO_2 nanotube array electrode	500 W high-pressure xenon short arc lamp (λ> 420 nm, 25.3 mW/cm^2, 0.6 V vs SCE), 80 min	*E. coli*, 100%	[4]
Ag/TiO_2 nanofiber membrane	Solar simulator (Xenon arc lamp, 100 mW/cm^2), 30 min	*E. coli*, 99.9%	[5]

References

[1] Yamaguchi, M.; Abe, H.; Ma, T.; et al. Bactericidal Activity of TiO_2 Nanotube Thin Films on Si by Photocatalytic Generation of Active Oxygen Species. *Langmuir* **2020**, *36*, 12668-12677.

[2] Mai, L.; Wang, D.; Zhang, S.; et al. Synthesis and Bactericidal Ability of Ag/TiO_2 Composite Films Deposited on Titanium Plate. *Appl. Surf. Sci*. **2010**, *257*, 974-978.

[3] Panda, A. B.; Gopikishan, S.; Mahapatra, S. K.; et al. Bactericidal Efficiency of Nanostructured Al-O/Ti-O Composite Thin Films Prepared by Dual Magnetron Reactive Co-Sputtering Technique. *Ceram. Int*. **2014**, *40,* 4681-4690.

[4] Hou, Y.; Li, X.; Zhao, Q.; et al. Role of Hydroxyl Radicals and Mechanism of Escherichia coli Inactivation on Ag/AgBr/TiO_2 Nanotube Array Electrode under Visible Light Irradiation. *Environ. Sci. Technol*. **2012**, *46*, 4042-4050.

[5] Liu, L.; Liu, Z.; Bai, H.; et al. Concurrent Filtration and Solar Photocatalytic Disinfection/Degradation Using High-Performance Ag/TiO_2 Nanofiber Membrane. *Water Res*. **2012**, *46*, 1101-1112.
